# Predictive Assessment of Cancer Center Catchment Area from Electronic Health Records

**DOI:** 10.3389/fpubh.2017.00303

**Published:** 2017-11-16

**Authors:** Luca Salmasi, Enrico Capobianco

**Affiliations:** ^1^Department of Political Science, University of Perugia, Perugia, Italy; ^2^Center for Computational Science, University of Miami, Coral Gables, FL, United States

**Keywords:** catchment area, cancer patients, multivariate adaptive regression splines, bootstrap, testing

## Abstract

Healthcare facilities (HF) may identify catchment areas (CA) by selecting criteria that depend on various factors. These refer to hospital activities, geographical definition, patient covariates, and more. The analyses that were traditionally pursued have a limiting factor in the consideration of only static conditions. Instead, some of the CA determinants involve influences occurring at both temporal and spatial scales. The study of CA in the cancer context means choosing between HF, usually divided into general hospitals versus oncological centers (OCs). In the CA context, electronic health records (EHRs) promise to be a valuable source of information, one driving the next-generation patient-driven clinical decision support systems. Among the challenges, digital health requires the re-definition of a role of stochastic modeling to deal with emerging complexities from data heterogeneity. To model CA with cancer EHR, we have chosen a computational framework centered on a logistic model, as a reference, and on a multivariate statistical approach. We also provided a battery of tests for CA assessment. Our results indicate that a more refined CA model’s structure yields superior discrimination power between health facilities. The increased significance was also visualized by comparative evaluations with *ad hoc* geo-localized maps. Notably, a cancer-specific spatial effect can be noticed, especially for breast cancer and through OCs. To mitigate the data distributional influences, bootstrap analysis was performed, and gains in some cancer-specific and spatially concentrated regions were obtained. Finally, when the temporal dynamics are assessed along a 3-year timeframe, negligible differential effects appear between predicted probabilities observed between standard critical values and bootstrapped values. In conclusion, for interpreting CA in terms of both spatial and temporal dynamics, sophisticated models are required. The one here proposed suggests that bootstrap can improve test accuracy. We recommend that evidences from stochastic modeling are merged with visual analytics, as this combination may be exploited by policy-makers in support to quantitative CA assessment.

## Introduction

The value of information [or VOI; (1)] is a concept still relatively unknown to the medical scientific community (2), but often empowered by precision medicine, is *de facto* a consequence of two interlinked aspects: a qualitative one, i.e., rescaling evidence-based medicine to a multidimensional structure of information, and a methodological one, i.e., integrating multiple dimensions. This idea finds application in several contexts, including public health tasks of determining and planning both quality and extent of health services to the population. Data are a valuable resource for such decision processes and are increasingly represented by electronic health records (EHRs). These types of data present analytical and computational complexities that vary depending on groups, communities, and sub-populations.

The aim of this work is to establish the relevance of EHR-driven approaches for the identification of catchment areas (CA) in relation to healthcare facilities (HF) (3). With CA one defines a geographical area whose services function as attractors for reference patient populations. A primary objective is to determine the probabilities of choosing between types of HF. Among the factors determining the choice, there are the accessibility to service and the volume of activities at the HF. Quite evidently, these are focal planning factors that policy-makers consider. However, due to the attention for diverse contexts depending on geographical reasons, an emergent risk is de-emphasizing the relevance of other factors, such as disease burden or socioeconomic indicators. Statistical inference methods have only partially considered such factors when models and systematic treatment have been proposed (4).

The complexity of assigning value to CA is high and includes VOI among other factors. In general, a main economic purpose is to estimate the utilization rate and performance in relation to the quality of service. VOI estimates the value of collecting additional data aimed to reduce decisional uncertainty. In our specific context, it is useful to assess CA decisions in a more objective way in support of choices about access to HF on the basis of cancer burden. To examine the expected value, a main consideration goes to the existence of barriers versus facilitators. We try to infer their possible existence for a target cancer population from real-world data available in the form of EHR and in relation with a differentiated healthcare offer. It is expected that local patient demand will vary in response to diversity of clinical practice depending on a series of variables. Even if we cannot clarify necessarily what is the best practice, we aim to indicate where the prevalence of one or another type of HF can be localized, considering all reference municipalities present in the territory covered by our data.

Assessment of the social impact would require a population-based estimate of disease burden. Beneath both economic and social purposes, there are several aspects that need to be examined. Traditionally, variables such as the distance from the facility or the road networks surrounding the facility have been studied, for instance, relatively to NYCLIX data (5) or the Montreal area (6). It was primarily shown that patients’ behavior is quite unconstrained. If patients “walk free” for healthcare matters, expectedly, in turn such diversity causes VOI fragmentation. The complexity of CA-driven interactions thus increases and is transferred to the observable patient flow. Being VOI recovery a priority at both patient and population scales, there are a few possible actions that EHRs may enable. For instance, critical is the need of inferring more accurately HF access. When the criterion of choice is centered on the disease, the interest might go to quantitative measures such as the cumulative case ratio (i.e., the ratio of the observed to the expected number of disease-related visits to the facility). This element has been considered in a recent study (7).

However, together with individual patients also the HF characteristics play a role. Moreover, both patient- and HF-driven influences are dynamic entities as they change with time, thus justifying the need for probabilistic CA studies. This idea indeed offers the rationale for our study. Clearly enough, over-/under-estimation of such influences can bring bias, limit the potential of the observational study, bring in confounders and information gaps. Among the factors determining explanatory power in models, there are location (poverty), demand and supply (for instance, number of attendances and number of beds, respectively), management issues, accessibility (waiting list), etc. (8). Notably, an important aspect associated with such factors is population deprivation, which stands out as a main variable for general investigation of social impact.

Healthcare problems from a CA perspective present complexities that can be reasonably framed within a systems’ inference approach in which important components are represented by actions and transformations from various subjects, combined with their determinations or results (9). Following this approach, our EHR-driven analyses are centered on a repository of cancer records of the Region of Umbria (Italy). Being the disease burden related to cancer, CA is defined with reference to the capacity of specialized oncological centers (OCs) versus more general hospitals (GH) to attract patients. As for the statistical methodology, we adopt a probabilistic baseline model versus a more refined multivariate one, both allowing predictive inference of correct decisions under uncertainty.

## Materials and Methods

### Data Structure and Covariates

Hospital discharge records were generated by both private clinics and public hospitals in the Region of Umbria (Italy). These data collect at the patient level information on hospital admissions for various diagnosis-related groups. Records include the prescribed therapy, the division of treatment, plus inter-division transfers. We selected only patients with a diagnosis of malignant cancer and followed up during 2007–2009, and five different cancer types (lung, bronchus, and trachea; breast; cervical; ovary; prostate, plus other residual malignant cancers), for female/male patients. The total number of observations is 35,962 (35,960 without missing values). We included cases with diagnosis of cancer according to the ICD-9-CM codes, addressing 8,740 (2007), 8,983 (2008), and 9,095 (2009) diagnosed/treated patients. Umbria, one of the 20 regions of Italy, has 894,762 inhabitants, approximately 1.5% of the Italian population. The potential generalizability of our approach may expand to cover >140,000 patients per year, accounting for the whole Italian territory.

Naturally enough, determining the covariate set is a key step. Thus, *X_ij_* is the covariate vector including both hospital- and municipality-related variables, and also variables measured at the individual level. With regard to hospital-related covariates, relevance is assigned to (a) a measure of the waiting time with respect to the HF (covariate named *waiting time*), measured as the number of days between the appointments’ reservations and the admissions; (b) the distance, measured by the Euclidean distance in kilometers between the centroid (a geometric center of a polygon) of municipality from the closest facility (covariate named *distance*). With regard to individual covariates, we controlled for (a) *gender*: male and female (ref. category); (b) *age class*: <45 (ref. category), 45–65, and >65; (c) *nationality*: Italian or foreign-born (ref. category); (d) *type of cancer*: the five stated before, and residual other malignant cancers (ref. category); (e) *presence of comorbidities*, according to the Charlson comorbidity index (CCI) score, calculated as in Ref. (10). Here, a score is assigned to each comorbidity on the basis of its degree of severity. The CCI score is calculated as the sum of individual scores (with respect to each comorbidity). The number of visits per year, from 1 (ref. category) to 5, was used as a categorical control variable.

Measures for patients’ economic status and education are missing. Therefore, to balance for this gap in spatial dimension, we calculated from census data for the year 2001 (source: the Italian Institute of Statistics) a deprivation index for the 92 municipalities of Umbria, (see Part A in Supplementary Material for further details). The index is obtained as the sum of standardized scores, then categorized on the basis of the quartiles of the observed score distribution [indicated, respectively, by *sed1* (ref. category), *sed2, sed3, sed4*] by increasing levels of socioeconomic deprivation. Notably, we established the dynamic aspect of our analysis through variables measuring cancer-specific time trends to capture variations in the choice of the alternative HF. Finally, we also included year- and quarter-specific dummy variables (2007, and the first quarter represent reference categories).

### Statistical Models

Considering the previous literature, namely, (A) generalized additive model and generalized additive mixed model employed with different reference CA and data sources (11) to capture non-linear relationships between covariates and random effects and to perform also spatial analysis as semi-parametric extensions of Eq. 1; (B) Bayesian models, but facing limitations with regard to the number of included population strata (12), two methodological approaches were here evaluated to define CA. The first approach is centered on a parametric logistic regression model employed to estimate the odds ratios (ORs) quantifying the probability of choosing a specialized OC versus GH (our reference category), given a set of selected covariates. Formally, our baseline model for an individual indexed by *i* is:

(1) $$log\left(\frac{p_{i}}{1-p_{i}}\right)=\alpha_{0}+\overset{J}{\underset{j=1}{\sum \;}}\beta_{j}X_{ij}+\epsilon_{i}.$$

Here, *p_i_* = Pr(*y_i_*) and *y_i_* is a dichotomous variable taking value 1 if an individual *i* chooses to access the OC and 0 otherwise. In this specification, our data were considered as cross-sectional but with no knowledge of the panel structure. Therefore, every time an individual is diagnosed or treated for cancer, this represents a new observation. Then, *p_i_* is the probability of choosing the OC and 1 − *p_i_* is the probability of choosing the GH. Naturally enough, determining the covariate set is a key step. Thus, *X_ij_* is the covariate vector including both hospital and municipality-related variables, and also variables measured at the individual level.

The second approach relies on a flexible non-parametric estimation of the probability of using the alternative HF, through the popular high-dimensional smoother *multivariable regression spline* (MARS) (13). This model generalizes univariate regression splines based on the adaptive construction of two-dimensional basis functions. In particular, the smoothing parameter for these variables is selected both individually and for their interactions (14). As a result, such flexible smoothers can estimate non-linear relationships between a dependent variable and a set of independent variables, and non-parametrically, thus regardless the underlying distributional relationships. Notably, the smoothly connected basis functions have support on a particular region in which the regression simplifies to a product of basic functions such as splines with knots at the boundaries of such regions, and whose inherent flexibility can handle both linear and non-linear forms. Following Ref. (15), we sketch the scaffold model for a generic outcome as follows:

(2) $$y_{i}=f\left(X_{i1},\ldots ,X_{ip}\right)+e_{i}=f\left(X_{ij}\right)+e_{i}.$$

As before, *y_i_* is the outcome (OC versus GH), *e_i_* is the error, and *X_ij_* is the vector of covariates at the individual, municipal and hospital levels. MARS utilizes splines *f* as the basis functions, i.e., piecewise polynomial functions. Here, only the piecewise linear function is selected according to max (0, *x* − *t*) and with a knot defined at value *t* (max(•)), meaning that only the positive part of (•) is used, or otherwise a zero value is assigned. The MARS model systematic component *f*(*X_ij_*) is a linear combination of basis functions and their interactions, expressed as follows:

(3) $$f\left(X_{ij}\right)=\beta_{0}+\overset{K}{\underset{k=1}{\sum }}\;\beta_{k}\lambda_{k}\left(X_{ij}\right)$$

for each λ*_k_* as a basis function, while β*_k_* are coefficients estimated regression-wise. First, note that unlike with the baseline model, the categorical variables are now included as continuous variables to exploit MARS flexibility. In particular, this was performed for age and the socioeconomic deprivation index. Instead of including interactions between cancer type and year, we left to a cancer-specific polynomial time trend to explain the quarterly and yearly variability levels. MARS is inherently prone to approximation rather than estimation, making its interpretation not trivial due to variability outsourced from the number of terms, the selected regions, and the estimates of the coefficients (16). To estimate this model, we used the “mvrs” routine implemented in STATA. The procedure implementing the variables selection step and the relevant parameters used to perform the procedure are described in Part B in Supplementary Material [see Ref. (17) for further details].

### Hypothesis Testing for CA Assignment

We evaluated the models performance on the basis of accuracy, sensitivity, specificity, and no information rate (NIR). From each model, we obtained the predicted individual probabilities of using the types of HF and an estimate of the average probability of access to each municipality as follows:

(4) $$E_{r}^{M}\left[{\hat{p}}_{i}\right]=\left(1/N_{M}\right)\overset{N_{M}}{\underset{i=1}{\sum }}{\hat{p}}_{i}$$

$E_{r}^{M}\left[{\hat{p}}_{i}\right]$ stands for the expected value of the predicted probabilities ${\hat{p}}_{i}$ of choosing OC for each of the municipalities *M*, with *M* = 1, …, 92. By contrast, $1-{\hat{p}}_{i}$ represents the predicted probability of choosing GH, while the probabilities are estimated from either logistic (*r* = 1) or MARS (*r* = 2) models. *N_M_* represents the number of cancer patients in each municipality. Moreover, we can also define:

(5) $$E_{r}^{M}\left[1-{\hat{p}}_{i}\right]=\left(1/N_{M}\right)\overset{N_{M}}{\underset{i=1}{\sum }}\left(1-{\hat{p}}_{i}\right).$$

The quantity in Eq. 4 can also be expressed by including time as:

(6) $$E_{r}^{M}\left[{\hat{p}}_{i}|T=t\right]=\left(1/N_{M,t}\right)\overset{N_{M,t}}{\underset{i=1}{\sum }}{\hat{p}}_{i}I_{t}\left(p_{i}\right)$$

for *t* = 2007 − 2009, and the expected value conditioned on the selected year in Eq. 6. Then, *I_t_*(*p*) is the indicator function taking unitary value if *t* is equal to the selected year. Finally, *N_M,t_* is the number of cancer patients in municipality *M* also indexed by *t*. We used these measures to define two methods that identify whether in any given municipality the CA refers to OC or GH. The first method is a naïve assignment on the basis of a simple comparison between the estimated average probabilities for the HF:

(7) $${\text{CA}}_{r,t}^{M}=\{\begin{array}{c} \text{OC if }E_{r}^{M}\;\left[{\hat{p}}_{i}\right]>0.5\\ \text{GH otherwise}. \end{array}$$

Defining CA in a dynamic way implies a use of estimated probabilities from any statistical model. However, a method may not uniquely identify a CA with OC or GH for a given municipality. In fact, close probabilities may stand for the two alternative facilities, making this identification less precise. To define a more efficient method for CA assignment, we tested whether the difference between the estimated probabilities of choosing OC versus GH are statistically different. Such comparison can be typically performed by using the *z*-scores under the assumption of a normal distribution.

Thus, we define the following hypothesis systems in relation with the possible CA assignment to either OC (Eq. 8) or GH (Eq. 8-bis):

(8) $$\{\begin{array}{c} H_{0}:E_{r}^{M}\;\left[{\hat{p}}_{i}\right]\leq 0.5\\ H_{1}:E_{r}^{M}\;\left[{\hat{p}}_{i}\right]>0.5 \end{array},$$

(8-bis) $$\{\begin{array}{c} H_{0}:E_{r}^{M}\;\left[{\hat{p}}_{i}\right]\geq 0.5\\ H_{1}:E_{r}^{M}\;\left[{\hat{p}}_{i}\right]<0.5 \end{array}.$$

If the system of Eq. 8 rejects the null hypothesis, the municipality M is assigned to OC, and similarly in the system of Eq. 8-bis the assignment goes to GH.

Modified hypothesis systems for OC (9) and GH (9-bis) test CA assignment through time, i.e., dynamically:

(9) $$\{\begin{array}{c} H_{0}:E_{r}^{M}\;\left[{\hat{p}}_{i}\text{|}T=t\right]=E_{r}^{M}\;\left[{\hat{p}}_{i}\text{|}T=t+s\right]\\ H_{1}:E_{r}^{M}\;\left[{\hat{p}}_{i}\text{|}T=t\right]\neq E_{r}^{M}\;\left[{\hat{p}}_{i}\text{|}T=t+s\right] \end{array},$$

(9-bis) $$\{\begin{array}{c} H_{0}:E_{r}^{M}\;\left[1-{\hat{p}}_{i}\text{|}T=t\right]\text{=}E_{r}^{M}\;\left[1-{\hat{p}}_{i}\text{|}T=t+s\right]\\ H_{1}:E_{r}^{M}\;\left[1-{\hat{p}}_{i}\text{|}T=t\right]\neq E_{r}^{M}\;\left[1-{\hat{p}}_{i}\text{|}T=\;t+s\right] \end{array}.$$

Then, the usual critical values can be used to assess whether the standardized difference between the quantities described in the null hypothesis in Eqs 8 and 8-bis, Eqs 9 and 9-bis are equal or different from 0. This way, the problem of CA assignment is assessed according to a formal statistical test rather than simply relying on the naïve criterion defined in Eq. 7.

### Bootstrap Analysis

The hypothesis tests presented before have relied on normality of the distribution of the predicted probabilities average. If this assumption is not satisfied, the usual critical values are no longer informative about significance levels. Therefore, we relax the normality assumption and implement a procedure to empirically estimate the critical values in an attempt to perform more accurate tests. Following Ref. (18), we define a parametric bootstrap procedure, summarized in Part C in Supplementary Material. To assess the validity of our procedure, we compared the estimated bootstrap empirical distributions with the normal distribution by means of the Doornik–Hansen test. Finally, the *z*-scores will be used to assess acceptance and rejection of hypothesis tests. The *z*-scores are defined as the signed number of SDs by which the value of an observation (or data point) is above the mean value of the value being observed or measured.

## Results

Our results are organized as follows. We report two tables centered on the performance of two different models applied to various types of cancers (relatively to the total, lung bronchus, trachea cancers: 29.22%; breast cancer: 37.04%; prostate cancer: 26.91%). Model-driven maps that cover the territory (reference CA) are then differentiated according to the HF. Finally, in support of the numerical analyses, we then report extensive graphical evidence with disaggregated cancer data, in particular (a) lung, bronchus, and trachea; (b) breast cancer; (c) prostate cancer.

Table 1 shows the results obtained from the application of the baseline model of Eq. 1. Covariates are listed together with the corresponding estimates for regression coefficients and SDs. In a dynamic perspective, we included 3 years, 2007 (reference category), 2008 and 2009, and we also controlled for visits at a quarter frequency each year. As most of covariates are significantly different from 0, i.e., our specification is quite informative on patients’ choices, explaining 20% of variability in hospital choice by the Pseudo *R*-squared index.

**Table 1:** Estimated odds ratio from logistic regression.

Covariates	Coef.	SE
Waiting	1.1358***	(0.003)
Distance	0.9540***	(0.001)
Male	1.1025***	(0.039)
Age class: 45–65	0.9424	(0.058)
Age class: >65	0.7236***	(0.043)
Italian nationality	1.6946***	(0.137)
Charlson comorbidity index score	1.1439***	(0.037)
2 visits per year	1.1092***	(0.038)
3 visits per year	1.1561**	(0.069)
4 visits per year	1.3640***	(0.152)
5 visits per year	1.4727**	(0.258)
SED_2_	1.1915***	(0.055)
SED_3_	1.1036**	(0.051)
SED_4_	2.7269***	(0.134)

Cancer-specific dummy	Yes	
Year-specific dummy	Yes	
Cancer year-specific dummy	Yes	
Quarter-specific dummy	Yes	
Observations	35,960	
Pseudo R-squared	0.20	

*Choosing oncological center versus general hospital for diagnosis/treatment of various types of cancers (lung, bronchus, and trachea; breast; cervical; ovary; prostate cancer, plus other malignancies). Reference year: 2007. Records range: 2007–2009*.

SEs in parentheses are clustered at the individual level, significant levels as follows: ***p < 0.01, **p < 0.05, *p < 0.1. The total number of observed cases is reported at the end. Our model also controls for type of cancer, year, and cancer-year associations with specific dummy variables, thus capturing differences in the choice of the facility that could derive from unobserved factors and confounders related to each of the variables or associations between them.

Looking at ORs, we find that longer waiting times drive patients’ choices toward OCs (OR = 1.1358, CI_95%_ = 1.1283–1.1417), but regarding the distance from the closest OCs (OR = 0.9540, CI_95%_ = 0.9521–0.9559), individuals living far from OC prefer to access GH. Men are more prone to choose OCs than women (OR = 1.1025, CI_95%_ = 1.0209–1.1895) and individuals with age >65 are 28% less likely to use an OC (OR = 0.7236, CI_95%_ = 0.6646–0.7866). Italians are also more likely to use OCs than foreign-born individuals (OR = 1.6946, CI_95%_ = 1.2951–2.2158) exactly as individuals with larger CCI scores (OR = 1.1439, CI_95%_ = 1.063–1.229). As for the number of visits, we highlight increasing ORs, from 1.1092 (CI_95%_ = 1.0294–1.1948) for patients with a yearly visit, to 1.4727 (CI_95%_ = 0.8878–2.4407) for patients with five visits. Finally, increasing levels of SED correspond to higher accesses to OC, as ORs go from 1.1915 (CI_95%_ = 1.0693–1.3266) to 2.7269 (CI_95%_ = 2.0963–3.5448) for patients living in municipalities with SED in the second and in the last quartile of the distribution of the index of socioeconomic deprivation.

Table 2 shows estimates from the MARS model, using a specification similar to the logistic regression model (Table 1). Here, though, non-linearities are handled with continuous covariates by a more flexible non-parametric approach. For instance, the covariate associated to waiting times is specified with an initial df = 8 (the optimal level estimated by MARS was 8) (see Part B in Supplementary Material for more details about the “mvrs” routine and the df parameter). The same approach was adopted to estimate the effect of distance from the closest OC, and here too we specified an initial level of df = 8, while reaching an optimal level of 6 after the MARS optimization procedure (Table 2). For other continuous variables like age, CCI score, number of visits to the hospital and SED index, we used an initial value of 3, since the initial variability of these variables was lower, thus requiring a lower degree of complexity. After MARS optimization, we obtained for the above variables df values of 3, 1, 1, and 3, respectively.

**Table 2:** Estimated odds ratio (OR) and degrees of freedom (df) for each multivariable regression spline (MARS) predictor.

Covariates	Coef.	SE	df		Final knot positions
			Initial	Final	
Waiting: β1	1.1307***	(0.002)	8	8	3.084 6.433 11.64 13.65 9.053 20.49 11.73
Waiting: β2	1.0748***	(0.002)			
Waiting: β3	0.8345***	(0.002)			
Waiting: β4	1.0559***	(0.002)			
Waiting: β5	0.9376***	(0.002)			
Waiting: β6	0.8662***	(0.002)			
Waiting: β7	1.0698***	(0.002)			
Waiting: β8	1.0073***	(0.002)			
Distance: β1	0.9056***	(0.002)	8	6	39.48 12.73 17.28 23.9 29.33
Distance: β2	0.9275***	(0.002)			
Distance: β3	1.0175***	(0.002)			
Distance: β4	0.9804***	(0.002)			
Distance: β5	1.0056***	(0.002)			
Distance: β6	0.9855***	(0.002)			
Male: β1	1.0111***	(0.004)	1	1	
Age: β1	0.9668***	(0.002)	3	3	75 63
Age: β2	1.0066***	(0.002)			
Age: β3	0.9933***	(0.002)			
Italian nationality: β1	1.0139	(0.010)	1	1	
Charlson comorbidity index score: β1	1.0192***	(0.005)	3	1	
Visits per year	1.0264***	(0.002)	3	1	
SED: β1	0.9618***	(0.002)	3	3	2 3
SED: β2	1.0220***	(0.002)			
SED: β3	0.9639***	(0.003)			
Cancer-specific dummy	Yes		3	1	
Lung, bronchus, and trachea cancer-specific trend	Yes		3	1	
Breast cancer-specific trend	Yes		3	1	
Cervical cancer-specific trend	Yes		3	1	
Ovary cancer-specific trend	Yes		3	1	
Prostate cancer-specific trend	Yes		3	2	
Observations	35,960				
*R*-squared	0.58				
Adj. *R*-squared	0.58				

*Choosing oncological center (OC) versus general hospital for diagnosis/treatment of various cancers (lung, bronchus, and trachea; breast; cervical; ovary; prostate cancer; other malignancies). Reference year: 2007. Records range: 2007–2009*.

*Before choosing the thresholds, we performed preliminary analysis to assess the degree reached by each covariate in a MARS model in the absence of other covariates, and then used the highest value in the final specification. The results are shown in Table 1, with regard to waiting times and distances for which we found ORs that are higher/lower than 1. Non-linearities may arise for distance because a simple measure of it between the municipality of residence and the closest OC does not account, say, for road accessibility or for the presence of public transportation in any municipality. Therefore, people living in distant municipalities may get an easier access to OCs if they live by a motorway or in a municipality endowed with a good public transportation infrastructure. The same type of argument may apply for waiting times. In fact, for unobserved factors as reputation or advices received by parents or general practitioners, one could be induced to use an OC rather than the easier general hospitals alternative. All these effects cannot be controlled for efficiency in the fully parametric specification, unless one includes a full set of waiting- and distance-specific dummy variables, imposing to these variables a certain polynomial behavior. However, both alternatives are indeed not efficient from the computational viewpoint: the former would imply the inclusion of a very large set of parameters, thus posing challenges in terms of generalizability of results; the latter would be based on discretional choice about the degree of the polynomial to be used. The interpretation of MARS coefficients for the other covariates is similar to that proposed here. However, it is worth noticing how in this specification we included cancer-specific time trends varying by year and quarter, rather than including interactions between cancer type and year and quarter as in the logistic specification, again to exploit MARS flexibility*.

***p < 0.1.

Note the goodness of the final result in terms of explained variability. In fact, the *R*-squared and adjusted-*R*-squared indicators are now around 60% of the total variability (Table 2) versus 20% in the logistic regression model (Table 1). Then, given the dichotomous nature of our dependent variable, we also included a measure of accuracy calculated as 1 minus the misclassification rate, which shows how many times each model misclassifies the outcome variable (Table 3). The improvement in accuracy is substantial, from 54.20% of misclassified cases with the intercept only logistic regression, to 68.63% after the inclusion of covariates, and finally to 81.08% when MARS with covariates is used. We also displayed information about specificity and sensitivity measures, together with NIR. Notice how the difference between accuracy and NIR informs us about the amount of cases correctly predicted by our model compared to the accuracy calculated with all observation assigned to the majority class. Again, the MARS model performs again quite well, showing an increase in accuracy of almost 27% points, whereas considering the logistic regression the difference is of only 14% points.

**Table 3:** Model comparisons.

Model	Accuracy	Specificity	Sensitivity	No information rate
Logistic regression (with covariates)	0.6863	0.5725	0.5222	0.5420
Multivariable regression spline (MARS)	0.8108	0.8270	0.7918	0.5420

*MARS’s flexibility, due to the recursive partitioning nature, assigns simple basis functions through (*x* − *t*)+ or (*t* − *x*)+ in which *t* is a knot point determined in the model training process. The model first uses the basis functions to fit the data, and then prunes them. The removal of the least informative knots affects the estimated parameters, referring to interactions between multiple covariates, and this way the overall complexity can be managed by inducing both model estimation and model selection. The resolution allowed by knots to fit to the data influences the smoothing level, and if the data are sparse the overall performance (and generalizability) can decrease despite its ability to adapt to heterogeneity, thus to community type data*.

**Figure 1:**
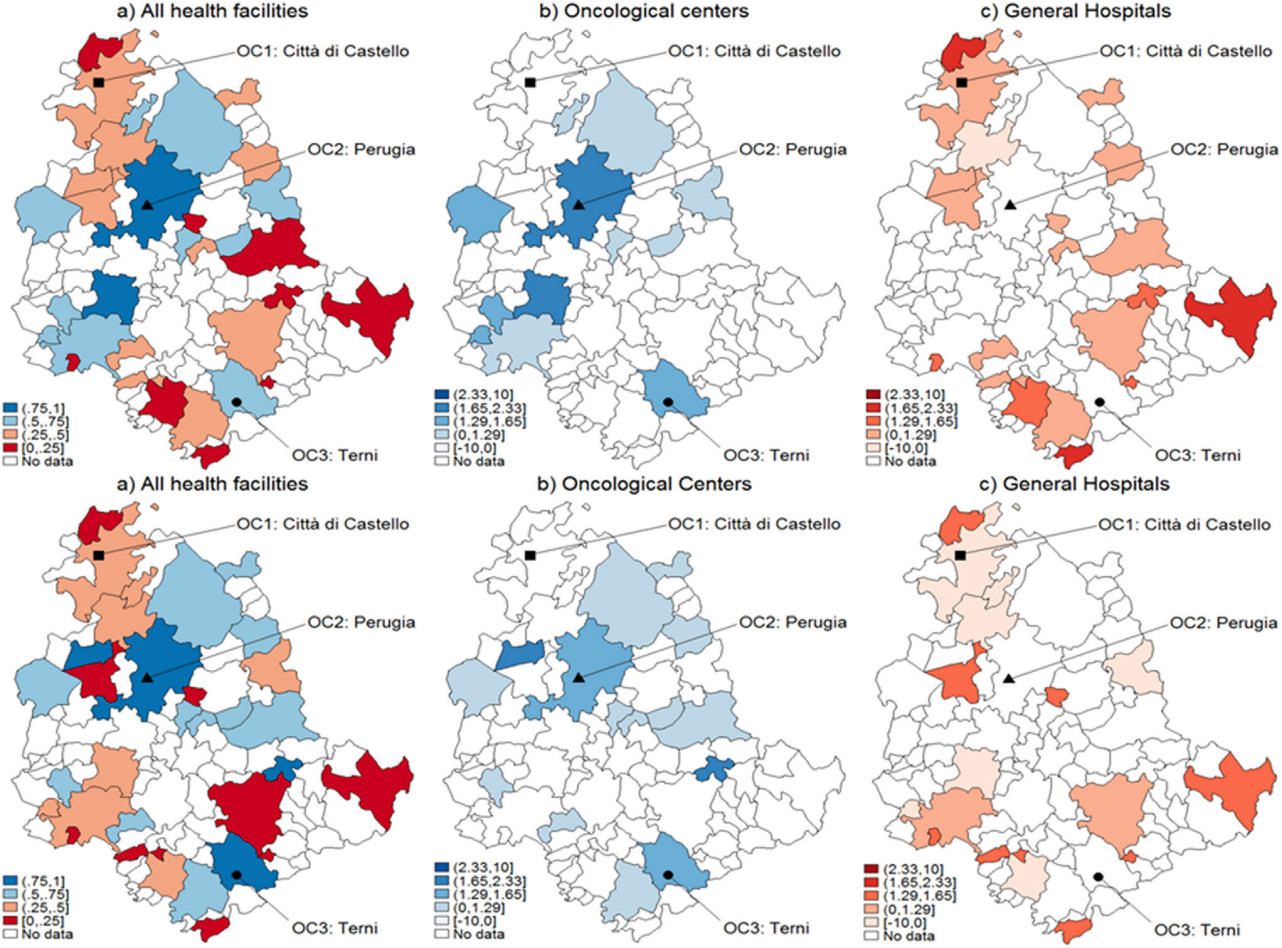
Catchment areas (lung, bronchus, and trachea cancers). Assignment by using predicted probabilities from the parametric logistic model (top row) and multivariable regression spline (MARS) (bottom row). Patients of the Region of Umbria (Italy): 2007–2009. All hospitals (left panels), oncological center (OC) (central panel), and general hospital (GH) (right panel). MARS appears overall as the best model in light of the more diffuse high significance spots in the map (left panels). Evidences indicate diverse assignments to OC and GH. Dark blue corresponds to a probability level between 75 and 100%, lighter spots represent decreasing levels of probabilities.

**Figure 2:**
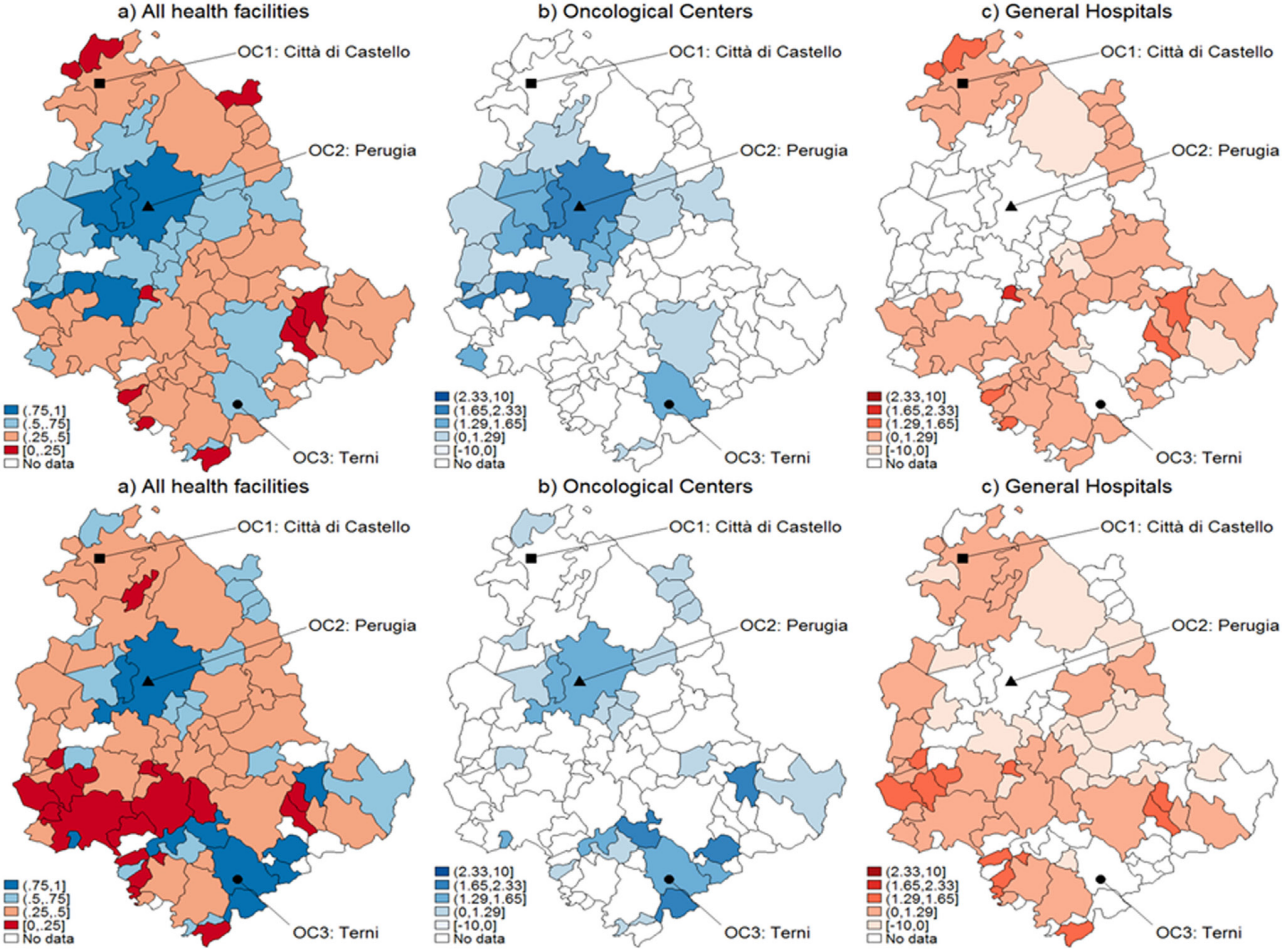
Catchment areas (breast cancer). Assignment by using predicted probabilities from the parametric logistic model (top row) and multivariable regression spline (MARS) (bottom row). Patients of the Region of Umbria (Italy): 2007–2009. All hospitals (left panels), oncological center (OC) (central panel), and general hospital (GH) (right panel). MARS appears overall as the best model in light of the more diffuse high significance spots in the map (left panels). Evidences indicate diverse assignments to OC and GH. Dark blue corresponds to a probability level between 75 and 100%, lighter spots represent decreasing levels of probabilities.

**Figure 3:**
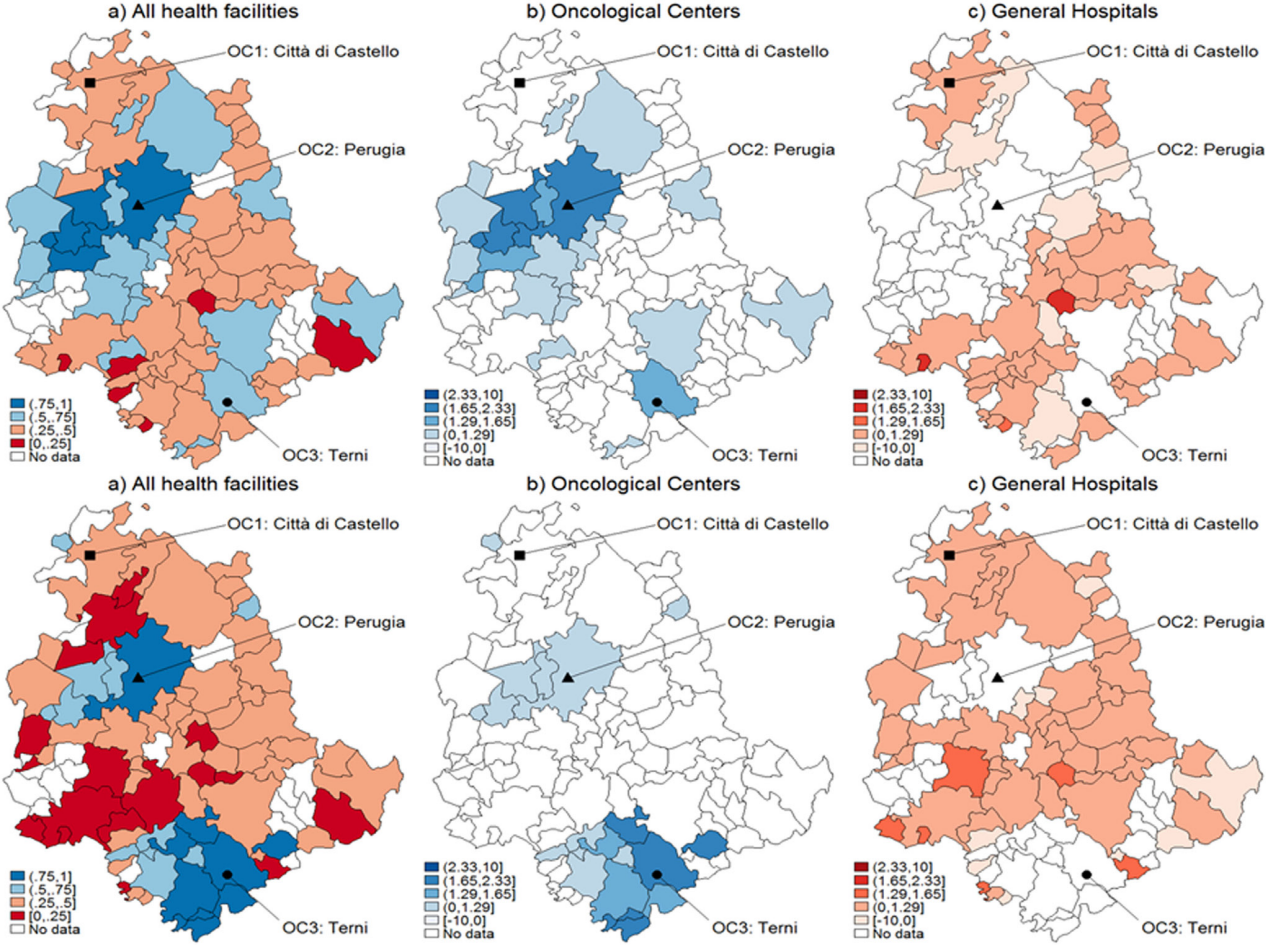
Catchment areas (prostate cancer). Assignment by using predicted probabilities from the parametric logistic model (top row) and multivariable regression spline (MARS) (bottom row). Patients of the Region of Umbria (Italy): 2007–2009. All hospitals (left panels), oncological center (OC) (central panel), and general hospital (GH) (right panel). MARS appears overall as the best model in light of the more diffuse high significance spots in the map (left panels). Evidences indicate diverse assignments to OC and GH. Dark blue corresponds to a probability level between 75 and 100%, lighter spots represent decreasing levels of probabilities.

With reference to CA assignment for selected types of cancer patients, the results of both logistic and MARS models appear in Figures 1–3, each subdivided in six panels representing the geographical variability in the probability of using OC versus GH for the whole territory (panel a) that results from the statistical hypothesis testing system presented (8) for OC (panel b), GH (panel c) with both logistic (upper plots) and MARS (lower plots) models. Looking at panel (a) of logistic versus MARS models, we can identify municipalities belonging to OC (GH) with blue (red) areas, while color intensity (darker spots) corresponds to higher (lower) levels of probability. From Figure 1 (including lung, trachea, and bronchus cancer), by considering the naïve assignment mechanism presented in Eq. 7, most municipalities belong to GH (20, about 22% of all municipalities and about 62.5% of municipalities with patients affected by this cancer), which appear dominant over OC that absorbs the remaining patients.

When looking at differential assignment with respect to the models, MARS assigns three more municipalities to OC. Similarly in Figures 2 and 3, with the assignment mechanism for breast and prostate cancer patients, respectively: more municipalities are assigned to GH (53 over 92 and 49 over 92, respectively). MARS assigns more municipalities to GH than the logistic model (from 53 to 57 and from 49 to 55, respectively). Due to its superior predictive ability, we kept only MARS for further analyses. Overall, we notice that independently on the model, a smaller number of municipalities is assigned to OC through the test.

**Figure 4:**
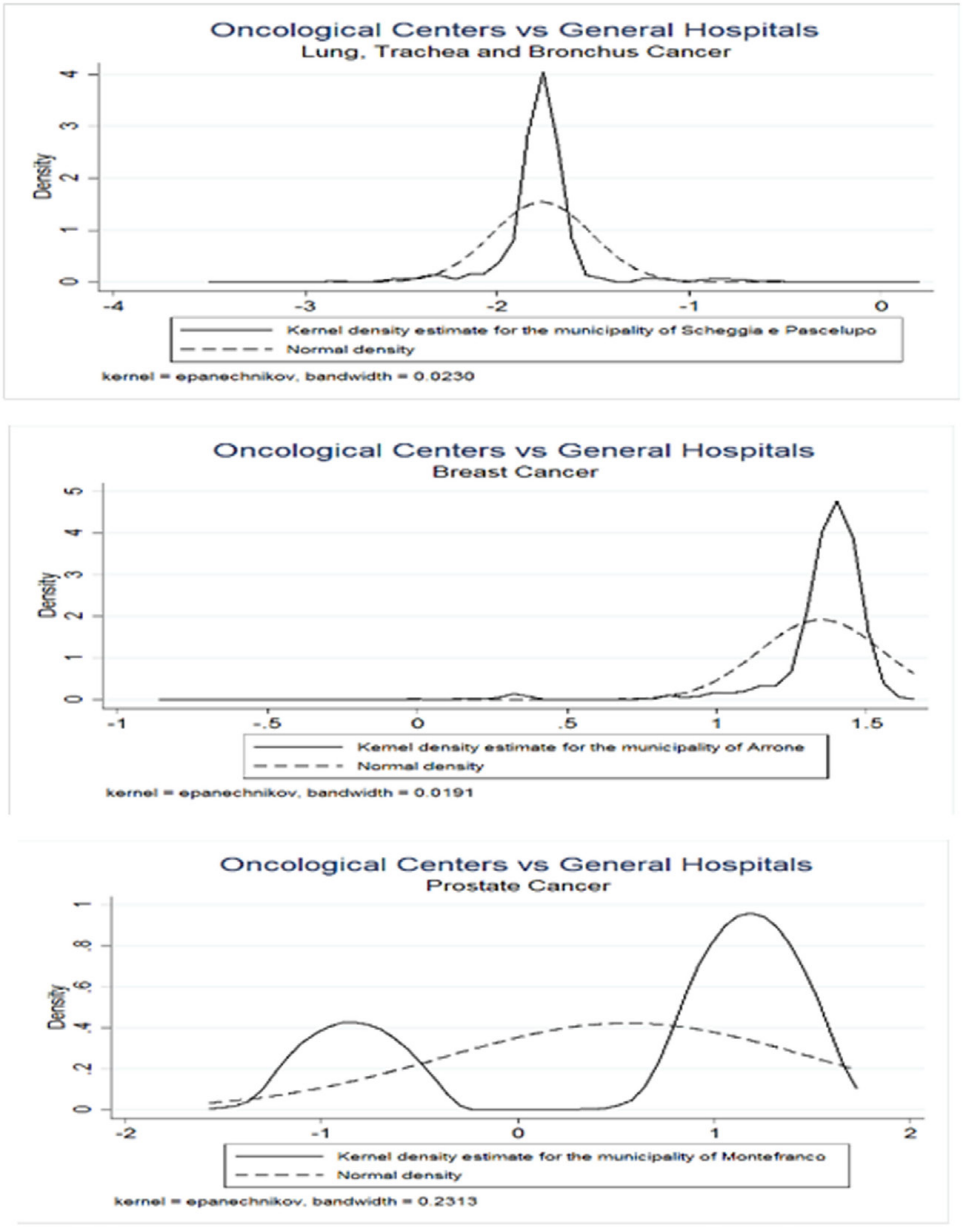
Bootstrap empirical distributions. Values on the *x*-axis represent *z*-scores. Municipalities were considered with greatest values of dissimilarity from Normal distribution, as indicated by the Doornik–Hansen test. Three types of cancers were considered, lung, bronchus, and trachea (upper panel); breast (middle panel); and prostate (lower panel).

Since the assumption of normality underlying the distribution of CA assignment probabilities to OC or GH may be not fulfilled, we propose a bootstrap method to obtain correct critical values for adjusting the comparisons between the averages of the predicted probabilities computed across municipalities. Table 4 presents the results from the Normality test for the empirical distributions across all municipalities. We observe that in all cancers only a minimal number of municipalities agrees with normality, i.e., 3.13, 3.80, and 6.7% are obtained with lung, trachea, and bronchus; breast and prostate cancer patients, respectively. Figure 4 presents the estimated empirical distributions (solid lines) for the three municipalities most dissimilar from the normal distribution, keeping the latter (dashed line) for comparison in each type of cancer. The estimated empirical distributions appear generally multimodal, heavy-tailed and asymmetric in each of the cancers.

**Table 4:** Normality tests for empirical bootstrap distributions, by municipality.

Municipality	Types of cancer					
	Lung, trachea, and bronchus cancer		Breast cancer		Prostate cancer	

Municipalities	Nr.	%	Nr.	%	Nr.	%
Normal distribution	1	0.0313	3	0.038	5	0.0676
Non-normal distribution	31	0.9688	76	0.962	69	0.9324
Total	32	1	79	1	74	1

**Figure 5:**
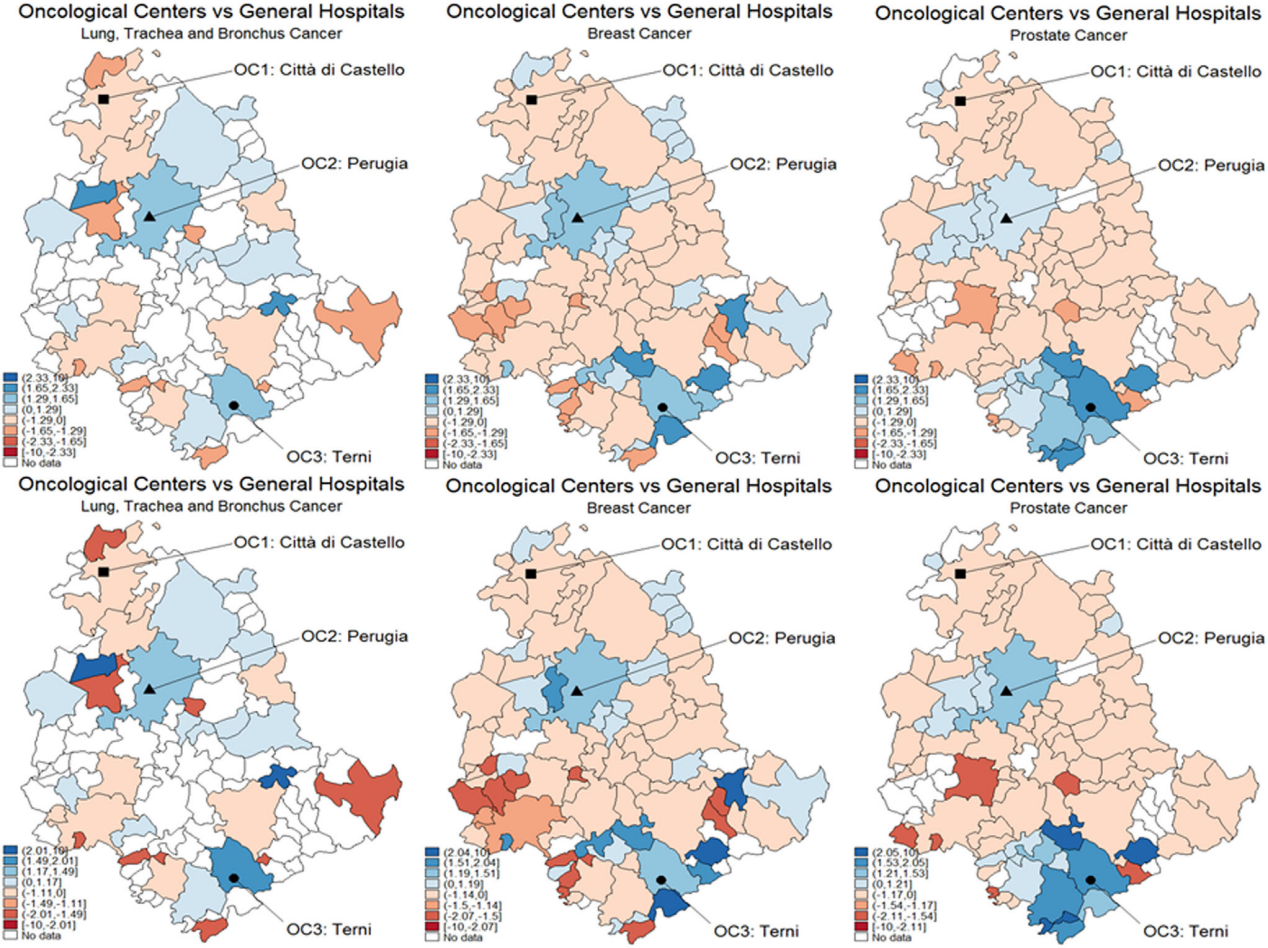
Test for catchment area assignment to OC or general hospital (GH) using *z*-scores. These were obtained as the difference of predicted probabilities from the multivariable regression spline (MARS) model for **(A)** lung, trachea, and bronchus; **(B)** breast **(C)** and prostate cancer patients of the Region of Umbria (2007–2009). Standard critical values at the upper panel; bootstrapped critical values at the lower panel. Dark blue corresponds to a positive difference significant at the 1% level, lighter spots represent decreasing levels of significance, 5 and 10%, respectively. Light blue corresponds to the interval [−1.645, 1.645], which means no significance. Lighter blue below the interval represents negative and significant differences, at the 10, 5, and 1%, respectively.

Figure 5 represents the graphical dispersion of the *z*-scores obtained as the standardized difference in the probabilities for all municipalities to be CA for OC versus GH, when considering all cancer types and the hypothesis systems Eqs 8 and 8-bis. In particular, both the upper and the lower panels show results obtained with MARS, the latter showing the bootstrap effects. Focusing on the hypothesis system of Eq. 8, the blue areas represent municipalities that are assigned to OC if $E_{r}^{M}\;\left[{\hat{p}}_{i}\right]$ is significantly larger than 0.5, whereas the red areas represent municipalities that are assigned to GH if $E_{r}^{M}\;\left[{\hat{p}}_{i}\right]$ is significantly lower than 0.5. For most municipalities, it is not possible to determine a clear assignment, since the *z*-scores lie mostly in the [−1.29, 1.29] interval, in which the differences are not significant. With prostate cancer, for instance, almost none of the municipalities assigned to OC from the naïve method in Figure 3 are confirmed by the new analysis. A clear assignment to OC is just visible for a few municipalities located at the South of Umbria. In general, this limitation indicates that the clear assignment shown in Figure 3 becomes fuzzier when we consider not only the absolute values of probabilities but also their differences. Then, a high competition degree between OC and GH is not evident from the naïve comparison. However, by using bootstrapped *p*-values some differences emerge, and while some municipalities assigned to OC are confirmed, other municipalities located Southeast are assigned to GH. This effect holds across cancers, as the bootstrap increases the number of municipalities assigned to each HF.

**Figure 6:**
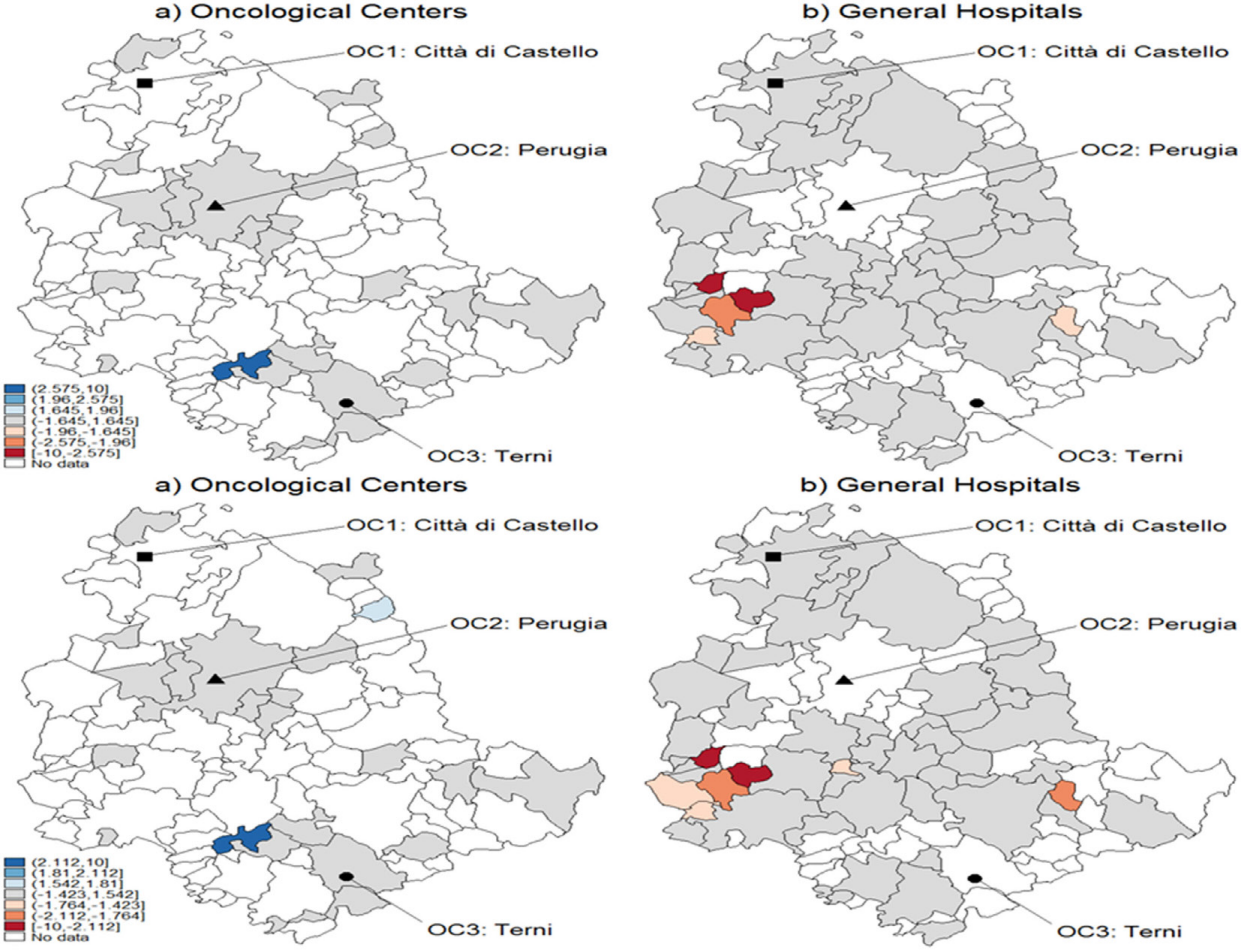
Test for CA temporal variation in breast cancer during 2007–2009 using *z*-scores. These were obtained as the difference of predicted probabilities from the multivariable regression spline (MARS) model. The example refers to breast cancer with standard critical values (upper panel) and with bootstrapped ones (lower panel). Patients of the Region of Umbria according to oncological center (OC) (left panel) and general hospital (GH) (right panel). Dark blue corresponds to a positive variation significant at the 1% level, while lighter spots represent decreasing levels of significance, 5 and 10%, respectively. Darker blue corresponds to the interval [−1.645, 1.645], which means no significance, and lighter blue below the interval represents negative and significant variations, at the 10, 5, and 1%, respectively.

Finally, in Figure 6, we show temporal effects for the breast cancer case, i.e., the standardized difference of the probabilities of being CA for both OC and GH in the interval 2007–2009. The patients affected by breast cancer are those subject to the highest temporal variability, according to the hypothesis systems Eqs 9 and 9-bis. In particular, the upper panel shows results obtained with MARS, and the lower panel shows the bootstrap effects. In many municipalities, the situation appears to be stable, i.e., no significant variations are noticed in the distribution of CA for these patients. Note that one municipality shows a significant increase in the probability of being CA for OC (panel a), whereas some municipalities decrease their probabilities of being CA for GH (panel b), especially those located southeast. From the lower panel with bootstrapped critical values, we can observe differences with regard to CA assignment. In fact, more significant temporal variations appear with OC, whereas the situation is less defined with GH.

## Discussion

Applied to the EHR cancer sources available to this CA study, the first conclusion is that the modeling task needs to refer to some considerable structure, here reflected into the superior performance of MARS over more basic reference models. A main statistical limitation remains beyond the choice of the model framework, namely that the CA estimated probabilities for each of the considered HF may follow any distribution, likewise the difference between such distributions which may be used in suitable tests. This limitation is in part mitigated by the choice of a non-parametric approach adaptive to the complexity induced by the presence of non-linearities and interactions between the variables in the model. To reduce the approximation errors, we also employed bootstrap and reconstructed the empirical distributions of the estimated probabilities, achieving more accurate acceptance/rejection regions for our tests. Assigning CA to OC versus GH through tests reveals effects specific to the selected cancers, even under a limited diffusion among municipalities. On the one hand, it is important to note the sensitivity of CA evidences to the type of cancer, particularly with MARS which shows better HF discrimination, and enables identifications of superior significance. The spatial effect induced by the cancer type is maximal with breast cancer compared to other cancers, especially in OC. The recourse to bootstrap boosts the significance toward discrimination between reference categories, but covers only cancer-specific spatially concentrated regions. On the other hand, modeling an increased number of municipalities may add power to the analysis and lead to improved test performances.

A second consideration is that when temporal effects are benchmarked against standard versus bootstrapped critical values, the expected differential effects for both OC versus GH appear negligibly present, something likely due to the time interval which is taken into consideration. In our study, this is only a 3-year interval, presumably too small. However, adjusting for distributional effects remains relevant to discriminating between spatial and temporal dynamics. Overall, it is safe to state that model assumptions can influence dynamic results but also that taking care of data size effects helps, as these could be responsible for the differentiated patterns observed through the number of patients examined in each cancer context. Another limiting factor inherent to models is overfitting, possible given the non-linearity of MARS and performance evaluation based only on observed data. However, the problem is at least mitigated by the fact that the data have a high observations/features ratio. Finally, and as a technical note, the degree of interaction allowed in the MARS model depends on the available algorithm. Our selected method specifies model interactions by including variables, for example, cancer-specific time trends capturing unobserved heterogeneity in the outcome variables.

The results here obtained are relevant to policy-makers in view of the possible generalization of the approach here manifested in its potential by a highly contextualized assessment (i.e., a few selected cancers, the municipalities of only one geographic/administrative region). Indeed, while OC are expected to absorb more cancer patients, economic resources that are not properly allocated may play a role. The presence of excessively long waiting lists or the localization of hospitals across the regional territory may induce individuals to choose GH. From a simple model comparison of point estimates of CA probabilities, OC would dominate over GH in terms of both diagnosis and treatment of cancer patients. However, a fuzzy picture emerges with more sophisticated models, and decisions based on hypothesis systems testing whether point estimates are statistically different. In fact, we are able to clearly assign CA to either GH or OC only for a fraction of municipalities, and we cannot assess whether such significant differences are persistent along time, or instead they just represent idiosyncratic shocks caused by transitory phenomena. Different models could be selected to integrate new covariates and allow for better treatment of heterogeneity at geographical population level. The dynamic assignment implies also the possibility to use the model with predictive purposes, especially toward territories facing critical care needs due to changes at either geographical or demographic levels.

With regard to demographics, it is expected that variations may affect target CA and also surrounding areas. Notably, recent efforts have been made to integrate accessibility and location–allocation models in geographic information systems, thus considering spatial planning (19, 20). Outcomes not necessarily related to HF access may include a better understanding of the level of performance of healthcare on the basis of the sustained HF costs. Also, measures could refer to use of person-specific treatment effects (21), following (22, 23), and recently (24), with these effects aggregated at the hospital level, and costs derived directly by hospitals’ accounting systems, say. This would be relevant to measure the performance of CA cost–benefit analyses when data disaggregation by HF (specialized versus general) is taken into account.

We conclude by considering that CA is subject to various principled analyses. A definition of cancer burden according to specific criteria appeared recently in a study from the California Cancer Registry (25). The study considered the number of cases and deaths, temporal trends, medical costs, risk factors, differences by ethnicity, and sex. The results indicated CA defined on the basis of case density subject to changes over time, and this motivates the study of selected cancer sites with increased specializations. Our statistical analyses consider observed cancer densities fluctuating over a limited time interval across a specific territory within two selected sites. Limitations are of course present, for instance, in the variable distance (in kilometers) measured between two points, the centroid of the municipality of residence and the centroid of the municipality with the closest hospital. Distance is generally used in empirical analysis to proxy accessibility, but other appropriate measures could be used. For instance, distance does not necessarily account with sufficient accuracy for time of travel, which could be longer when accessibility is restricted by physical constraints. In general, increased travel burden is associated with survival disadvantages for dispersed populations (26).

## Data Availability and Software

Hospital discharge records were generated by both private clinics and public hospitals in the Region of Umbria (Italy) from anonymized patient records at the following source: http://www.salute.gov.it/portale/temi/p2_4.jsp?area=ricoveriOspedalieri.

## Software Source

Stata, mvrs. http://www.stata-journal.com/sjpdf.html?\articlenum=st0120-.

## Author Contributions

The authors have equally contributed to writing the paper and deciding the methodological approach. LS performed computations. EC guided through literature review on concept and methodologies.

## Conflict of Interest Statement

The authors declare that the research was conducted in the absence of any commercial or financial relationships that could be construed as a potential conflict of interest.
