# Supplementary material for: Predictive Assessment of Cancer Center Catchment Area from Electronic Health Records
Source: Front Public Health. 2017 Nov 16;5:303. doi: 10.3389/fpubh.2017.00303 (PMC5696335; doi:10.3389/fpubh.2017.00303)
Supplement: Supplementary file 1 [file data_sheet_1.pdf]

Supplementary Material.

A. Deprivation Index.

The deprivation index is defined using five variables, all suitably standardized. The variables are the percentage of individuals who did not complete compulsory education, the percentage of unemployed individuals or looking for their first job, the number of individuals per dwelling, the percentage of rented accommodation, the percentage of single parents living with at least one child. Standardization is applied as follows:

$z_i=(x_i- \mu_x)/\sigma_x$,

where z_i, with i=1,…,5 is the standardized i-th component of the deprivation index, x_i is the raw i-th index, and μ_x and σ_x are the means and standard deviations of raw index i. The index is thus obtained as the sum of the standardized scores, then categorized on the basis of the quartiles of the observed score distribution (indicated respectively by sed1, sed2, sed3, sed4). We used the first quartile (sed1) as the reference category; the other categories indicate increasing levels of socio-economic deprivation.

B. The Multivariate Adaptive Regression Splines (MVRS) model

The mvrs procedure combines the selection of simplified spline functions with backward elimination of weak predictors. The algorithm is implemented by a closed-test procedure (Marcus, Peritz, and Gabriel 1976), a sequence of tests designed to maintain the overall type I error probability at a pre-specified α nominal level, such as 0.05. The quantity α is the key determinant of the complexity (in dimension, and therefore in shape) of a selected function. Initially, the most complex permitted regression splice (RS) model is chosen, which is determined by the df assigned to the RS function. The df are quantified by m + 1, where m is the maximum number of knots to be considered, and m = 0 means the linear function.

By default, “mvrs” takes m = 3 and df = 4. It is possible to select different df through the df () option.

Example.

Let us call the most complex model Mm and the linear function, M0. First, model Mm is compared with the null model (omitting x), using a $\chi 2$ test with m + 1 df. If the test is not significant at the α level, the procedure stops and x is eliminated. Otherwise, the algorithm proceeds and compares the fit of Mm with that of M0, with df moved to m. If the deviance difference is not significant at the α level, M0 is chosen by default, and the algorithm stops.

Now consider the m possible RS models using just one of the m available knots. The best fitting of these models, say M1, is found and compared with Mm. If Mm does not fit significantly better than M1 at the α level, there is no evidence that the more complex model is needed, so model M1 is accepted and the algorithm stops. Otherwise, M1 is augmented with each of the remaining m −

1 knots in turn; the best-fitting model, M2, is found; and M2 is compared with Mm. The procedure continues in this fashion until either a test is non-significant, such that the procedure stops, or all the tests are significant, in which case the model Mm is the final choice.

All the tests are based on the χ2 statistics from deviance (−2 × log likelihood) differences. Each predictor is considered in turn, with the functions and inclusion/exclusion status of all other variables temporarily fixed. Variables are considered in decreasing order of statistical significance in a full linear model. The algorithm cycles over each predictor repeatedly in the same order, changing the model according to the results of the tests of individual variables—see "Selecting an RS function for one predictor".

The process stops when there is no further change in the variables included in the model and in the spline functions (knots) chosen for each continuous variable.

C: Bootstrap procedure.

We followed the procedure proposed by Efron &Tibshirani, (1993).

i) For each municipality M, a random sample of the population of size n_M is drawn with replacement;

ii) n_M is chosen as a fixed percentage of the original sample size N_M.

iii) The bootstrapped samples s_M have size covering 30% of each N_M.

iv) At each iteration, the following statistics of interest are computed, depending on weather we are interested in testing hypothesis systems (8, 8-bis) or (9, 9-bis): $E_r^{Ms*}[\hat{p}_i]$, $E_r^{Ms*}[1-\hat{p}_i]$, $E_r^{Ms*}[\hat{p}_i|T=t]$ and $E_r^{Ms*}[\hat{p}_i|T=t+s]$. Then at each iteration we evaluate a statistic of interest, e.g.: $t(E_r^{Ms*}[\hat{p}_i]) = E_r^{Ms*}[\hat{p}_i]-0.5$, with s = 1,…,S representing bootstrap replications.

v) The bootstrap p-values are computed as:

$$\hat{p} = \#\{t(E_r^{Ms*}) \geq t_{obs}\}/S, \qquad (12)$$

with S as the number of bootstrap replications, 1,000 in our case, and t_{obs} as the observed statistic on the original dataset.

Supplementary References.

Ruth, M, Peritz, E. Gabriel, K.R. On closed testing procedures with special reference to ordered analysis of variance. Biometrika, 1976; 63(3): 655-660. https://doi.org/10.1093/biomet/63.3.655

Efron B, Tibshirani RJ (1993). An Introduction to the Bootstrap. Chapman & Hall, Inc..
